# Mast Cells Resting-Related Prognostic Signature in Hepatocellular Carcinoma

**DOI:** 10.1155/2021/4614257

**Published:** 2021-11-18

**Authors:** Hao Zhang, Lin Sun, Xiao Hu

**Affiliations:** ^1^Department of Hepatobiliary Pancreatic Surgery, The Affiliated Hospital of Qingdao University, Qingdao, Shandong, China; ^2^Department of ICU, The Affiliated Hospital of Qingdao University, Qingdao, Shandong, China

## Abstract

The immune microenvironment of liver cancer is of great significance for the treatment of liver cancer. After evaluating the content of mast cells resting in the transcriptome data of The Cancer Genome Atlas database by CIBERSORT analysis, this study aimed to group the samples according to the content of mast cells resting in different samples to find the differentially expressed genes in the two groups. Significant prognostic differences were found between high and low mast cells resting infiltration groups. The prognostic model was constructed according to the differentially expressed genes. The model was validated using external independent datasets. The results revealed that the constructed model was reliable. It could well distinguish the prognostic differences of patients in different characteristic groups. The high-risk group was mainly concentrated in metabolic pathways. The risk score of this model was closely related to some immune cells, immune function, and immune checkpoints. Therefore, this model may provide new ideas for immunotherapy of hepatocellular carcinoma.

## 1. Introduction

Hepatocellular carcinoma (HCC) is highly malignant liver cancer and a major health problem worldwide. Its prognosis is poor because of its highly metastatic and recurring nature. Improvement in the prognosis of liver cancer has become the goal of many researchers. Bucci et al. found that the epidemiology of HCC was changing [1]. In the past few years, drug-centered treatment has dominated, but the resistance of liver cancer to drugs has forced researchers to find other treatments. Recent studies found that the tumor microenvironment in patient tissues played an important role in supporting tumor cells. Among them, the immune microenvironment of liver cancer has been widely studied because of its complexity and particularity. Mast cells resting is particularly important in treating cancer because mast cells have a unique developmental, phenotypic, and functional plasticity. They participate in tissue homeostasis by constantly sampling the microenvironment [2]. Li et al. found that aberrant activation of mast cells and CD4+ memory T cells played crucial roles in cigarette smoking-induced immune dysfunction in the lung, which is important in tumor development and progression [3]. Walczak-Drzewiecka et al. found that hypoxia-inducible factor 1 alpha was upregulated in activated mast cells [4].

At present, a considerable number of studies have found that some genes affected the prognosis of patients with cancer. Yao et al. found that SNHG6 played an oncogenic role in colorectal cancer and is a prognostic biomarker [5]. Hlavac et al. found that the genetic variation of ATP-binding cassette was a prognostic marker in breast cancer [6]. Anwar et al. found that the T-cell factor-4 was a molecular target in the prognosis of sporadic colorectal cancer [7]. In recent years, a large number of studies have been devoted to finding multiple gene combinations to jointly predict the prognosis of patients with tumors. Chen et al. found lymph node metastasis-related key miRNA signatures in cervical cancer [8]. Shen et al. found the immune-related long noncoding RNA prognostic signature for breast cancer [9]. Liu et al. found a tumor immune microenvironment-related prognostic signature in epithelial ovarian cancer [10].

This study grouped genes related to high and low infiltration of mast cells resting according to the infiltration of immune cells in HCC. A prognostic model for these genes was constructed, and the model was deeply analyzed and discussed.

## 2. Materials and Methods

### 2.1. Data Download

The transcriptome data of HCC downloaded from The Cancer Genome Atlas (TCGA, https://tcga-data.nci.nih.gov/tcga/) were used to construct the model. The transcriptome data from Gene Expression Omnibus (GEO, https://www.ncbi.nlm.nih.gov/geo/) and the International Cancer Genome Consortium (ICGC, https://dcc.icgc.org/) were used to verify the reliability of the model. The corresponding clinical data were downloaded from the respective databases. The genes were annotated by gene transfer format files from Ensembl (https://asia.ensembl.org).

### 2.2. Construction and Validation of the Model

The genes used to construct the model were screened by the “limma” package (https://bioconductor.org/packages/limma/) in R software (4.0.0). The Cox hazard analysis and Lasso regression were used to analyze the genes combined with the prognosis of patients with “survival” (https://cran.r-project.org/package=survival), “glmnet” (https://cran.r-project.org/package=glmnet), and “survminer” (https://cran.r-project.org/package=survminer) packages. The survival curve and the receiver operating characteristic curve (ROC) were drawn using the “survivalROC” and “survival” packages.

### 2.3. Gene Set Enrichment Analysis

The Gene Set Enrichment Analysis (GSEA) was used to find meaningful biological characteristics in high- and low-risk groups of the TCGA cohort. An annotated gene set file (c2.cp.kegg.v7.0.symbols.gmt) was selected as the reference. The filter condition was false discovery rate (FDR) *q*-val <0.05.

### 2.4. Analysis of Immune Cells

The gene transcriptome data were used to estimate the content of multiple immune cells infiltrated in tumors, and the “StromalScore,” “ImmuneScore,” and “ESTIMATEScore” of each sample were found using the CIBERSORT analysis and “estimate” package. The “GSVA” and “GSEABase” packages were used for ssGSEA analysis and immune characteristic analysis of each patient. The correlation analysis of index was completed using the Spearman test.

## 3. Result

### 3.1. Generation of a Prognostic Model in the TCGA Cohort

**Figure 1:**
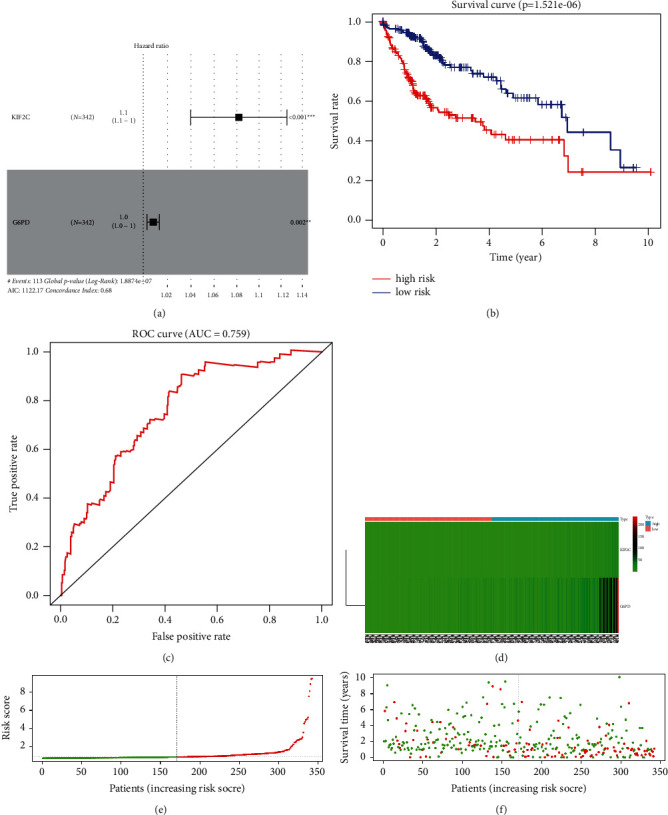
Generation of the prognostic model. (a) Forest map of multivariate Cox hazard analysis. (b) Prognostic characteristics between high- and low-risk groups. (c) ROC curves at 1 year in the TCGA cohort. (d) Expression of the two genes in the high- and low-risk groups. (e, f) Survival rates of patients with different riskScores.

A total of 1113 significant expression difference genes were found between the high-density mast cell group (86) and the low-density mast cell group of patients with HCC (87). The follow-up information of the patients was collected, and among the genes related to mast cell infiltration, 150 genes with the ability to significantly affect patients' survival were selected using univariate Cox hazard analysis. The number of genes was narrowed down, and finally, two genes were selected to optimize the model by Lasso regression and multivariate Cox hazard analysis (Figure 1(a)). The riskScore of each sample was calculated using the formula riskScore = KIF2C × 0.0786 + G6PD × 0.0082. The high- and low-risk groups were distinguished by the median of riskScore. The area under the ROC curve (AUC) was 0.759 at 1 year (Figure 1(b)). The survival status of the patients was significantly different between high- and low-risk groups (Figure 1(c)). The heatmap showed that the expression of KIF2C in the high-risk group was significantly higher compared with that in the low-risk group. The same trend was observed for G6PD (Figure 1(d)). The risk of death in patients with HCC increased with the increase in the riskScore (Figures 1(e) and 1(f)).

### 3.2. Verification of Model Reliability in Two Centers

**Figure 2:**
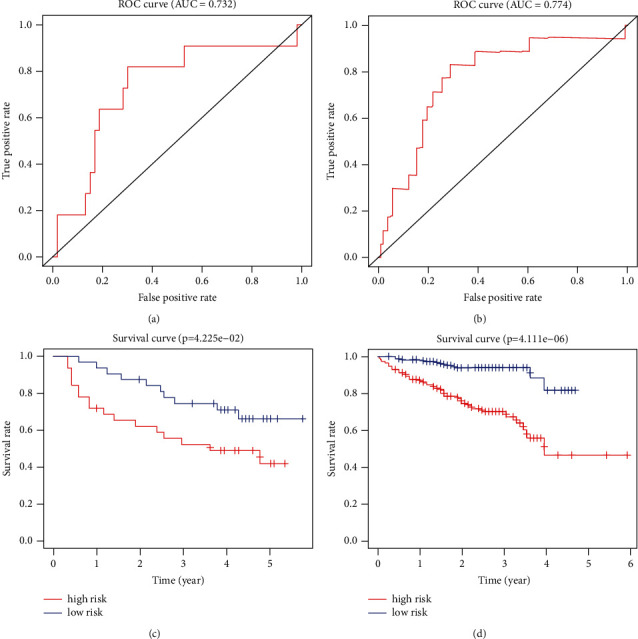
Verification of model reliability. (a) ROC curve at 1 year in the GSE14520 cohort. (b) Comparison of the survival status between different groups in the GSE14520 cohort. (c) ROC curve at 1 year in the ICGC cohort. (d) Comparison of the survival status between different groups in the ICGC cohort.

The AUC value was 0.732 at 1 year (Figure 2(a)), and the prognosis of patients in the high- and low-risk groups was significantly different (Figure 2(b)) in the GSE116774 cohort. The AUC value was 0.774 at 1 year (Figure 2(c)), and the prognosis of patients in the high- and low-risk groups was significantly different (Figure 2(d)) in the ICGC cohort.

### 3.3. riskScore Was an Independent Prognostic Indicator

**Figure 3:**
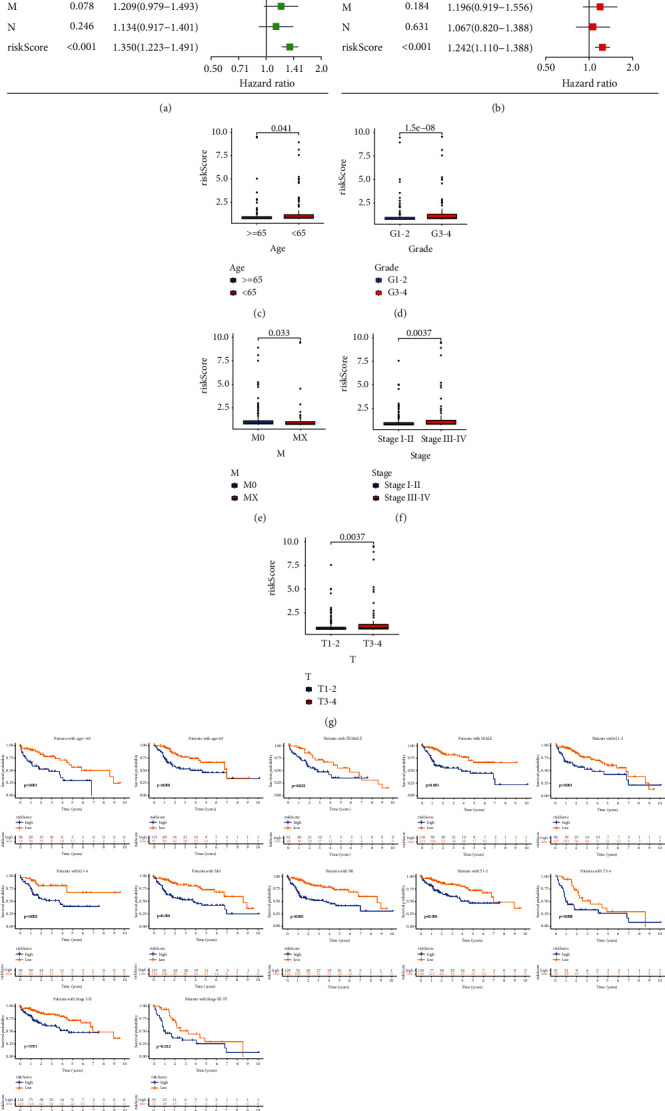
Relationship of the riskScore and clinicopathological features. Univariate Cox hazard analysis (a) and multivariate Cox hazard analysis (b) of patient's features. The distribution of the riskScore in different age (c), grade (d), M stage (e), clinical stage (f), and T stage (g) groups had significant differences. (h) Predicting the survival of patients in different age, gender, histological grade, M0, N0, clinical stage, and T stage groups by the riskScore.

The relationship between the constructed model and clinicopathological characteristics (age, gender, histological grade, clinical stage, and tumor-node-metastasis stage) was analyzed. The forest maps of univariate and multivariate Cox hazard analyses about clinicopathological features showed that the *P* value of the clinical stage, T stage, and riskScore was less than 0.001 and the hazard ratio was more than 1 (Figure 3(a)), and the *P* value of the riskScore was less than 0.05 and the hazard ratio was more than 1 (Figure 3(b)). The riskScore in different age, histological grade, M stage, clinical stage, and T stage groups had significant differences (Figures 3(c)–3(g)). The prognosis of different riskScore groups in different age, gender, histological grade, M0, N0, clinical stage, and T stage groups had significant differences (Figure 3(h)).

### 3.4. GSEA of Different riskScore Groups

**Figure 4:**
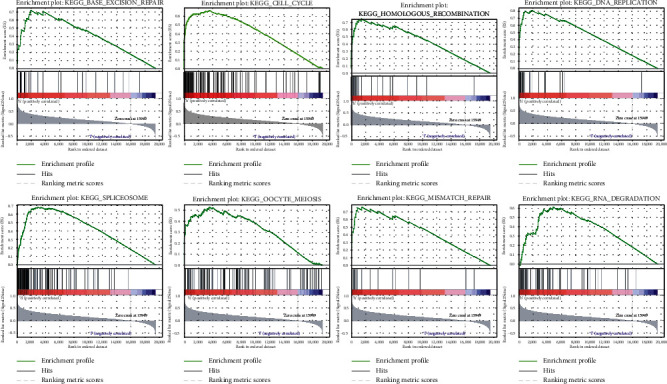
Gene sets of the high-risk group.

In the high-risk group, eight gene sets were found (FDR *q*-val < 0.05): BASE_EXCISION_REPAIR, CELL_CYCLE, HOMOLOGOUS_RECOMBINATION, DNA_REPLICATION, SPLICEOSOME, OOCYTE_MEIOSIS, MISMATCH_REPAIR, and RNA_DEGRADATION (Figure 4). Metabolic reprogramming was closely associated with patients in the high-risk group. In the low-risk group, 12 gene sets were found (FDR *q*-val < 0.05).

### 3.5. riskScore and Immune Cells

**Figure 5:**
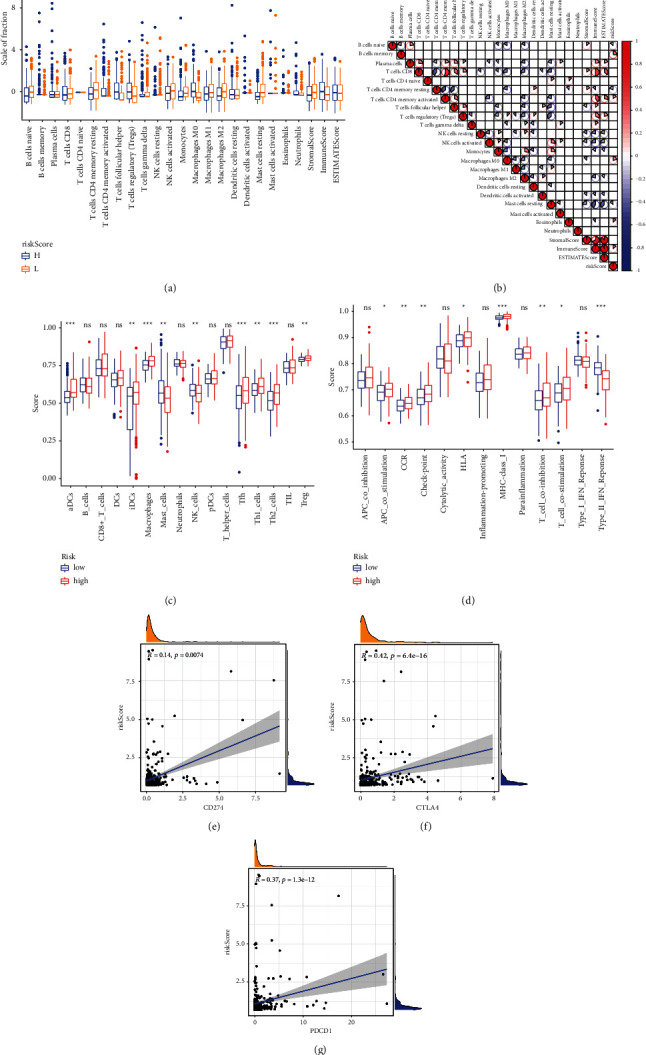
Model constructed in this study and immune cells. (a) Differences in immune cells between different groups. (b) Correlation between immune cells and the riskScore. (c, d) ssGSEA analysis of different riskScore groups. (e–g) Correlation between the riskScore and immune checkpoint.

Significant differences were found in dendritic cell (DC) resting density and mast cell resting density between high- and low-risk groups (Figure 5(a)). A significant correlation was found between the riskScore and immune cells (Figure 5(b)). No significant difference was found in the content of B cells, CD8+ T cells, DCs, neutrophils, plasmacytoid DCs, T-helper cells, and tumor-infiltrating lymphocytes in the high- and low-risk groups; antigen-presenting cell coinhibition, cytolytic activity, inflammation promotion, parainflammation, and type 1 interferon response were significant between the two groups by ssGSEA analysis (Figures 5(c) and 5(d)). A significant positive correlation was found between the riskScore and immune checkpoint (CD274, CTLA4, and PDCD1) (Figures 5(e)–5(g)).

## 4. Discussion

Liver cancer has been widely studied by clinicians and researchers because of its relatively high incidence rate and poor prognosis. A large number of experiments have been carried out and great progress has been made in the research of liver cancer. The popularity of high-throughput sequencing technology has led researchers to explore liver cancer from the perspective of genes, clarifying the biological role of a large number of genes in liver cancer.

The immune microenvironment of tumors can have a significant impact on the biological behavior of tumors. Therefore, the exploration of the immune microenvironment is particularly important. The infiltration density of mast cells in HCC and intrahepatic cholangiocarcinoma was found to be significantly higher than that in normal liver tissue [11]. It suggests that mast cells may play an important role in tumor immunity. It was found that high mast cell infiltration was associated with poor prognosis in patients with HCC [12]. Grizzi et al. found that mast cells may be considered necessary in the transition from sinusoidal to capillary-type endothelial cells and HCC growth [13]. Granito et al. studied that CD4+ CD25+ Foxp3 regulatory T cells could be induced by tumor-associated macrophages (by secreting interleukin-10) and indirectly support tumor growth and progression [14].

The model constructed in this study could significantly distinguish the difference in mast cell beauty in high- and low-risk groups, and the risk score negatively correlated with the content of mast cells, which was an exciting result. This model was constructed using two genes, which were found to play an important role in many tumors. Wei et al. found that KIF2C was a novel link between Wnt/beta-catenin and mechanistic target of rapamycin complex 1 signaling in HCC [15]. Zhu et al. found that KIF2C was important in regulating DNA double-strand break dynamics and repair [16]. Hong et al. found that phosphatase and tensin homolog antagonized G6PD pre-mRNA splicing, which contributed to hepatocarcinogenesis [17]. Rao et al. found that G6PD promoted tumor growth [18]. Ghergurovich et al. found that G6PD dehydrogenase was not important for K-Ras-driven tumor growth or metastasis [19].

The prognostic model constructed in this study was verified in two independent databases, which proved the reliability of the model. Only two genes were present in the model, which reduced the cost. The model might be closely related to many immune indexes, significantly improving the value of the model.

## Data Availability

The datasets used and/or analyzed during the present study are available from the corresponding author on reasonable request.
